# Lipocalin-mediated hydrophobic mismatch as a mechanism for sustained nonphotochemical quenching

**DOI:** 10.1093/plphys/kiag055

**Published:** 2026-03-03

**Authors:** Alexander V Ruban

**Affiliations:** Department of Biochemistry, Queen Mary University of London, London, UK

## Abstract

The plastid lipocalin sustains nonphotochemical quenching by stabilizing hydrophobic mismatch between the major light harvesting complex and the thylakoid membrane.

Dear Editor,

Nonphotochemical quenching (NPQ) plays a central role in protecting photosystem II (PSII) from excess excitation. NPQ complements both biochemical regulation of electron transport and structural adaptability of the thylakoid membrane (van Amerongen and Croce 2025). Although the rapidly inducible qE component is well characterized, the mechanistic basis of sustained NPQ (qH), which persists long after ΔpH has relaxed, remains less understood. Genetic studies have identified the plastid lipocalin (LCNP) as essential for qH, with its activity antagonized by the membrane-associated protein SOQ1 (Malnoë et al. 2018; Amstutz et al. 2020). Yet the biophysical mechanism by which a lipid-binding protein maintains a long-lived quenching state has remained elusive. Recently, the major light harvesting complex II (LHCII) has been proposed to be a site for qH (Bru et al. 2022). Here, I propose a mechanistic model in which LCNP stabilizes hydrophobic mismatch between the major LHCII and the surrounding thylakoid membrane, thereby converting a transient qE-like conformation into a metastable dissipative state characteristic of qH. This model integrates 3 established but previously unconnected observations: (i) the intrinsic sensitivity of LHCII to membrane lateral pressure and bilayer packing (Tietz et al. 2020); (ii) the finding that qE can arise from hydrophobic mismatch between LHCII helices and the thylakoid membrane (Wilson et al. 2024); and (iii) the biochemical ability of LCNP to bind hydrophobic ligands and associate with membrane surfaces, altering local packing properties (Lingwood 2014). Biophysical evidence supports the idea that membrane–protein mechanical interactions modulate chlorophyll energy dissipation in LHCII (Horton et al. 1996; Johnson et al. 2011). Changes in ΔpH, xanthophyll geometry, or lipid lateral pressure can shift the equilibrium between fluorescent and quenched conformational states. Wilson et al. (2024) demonstrated that qE can be reproduced as a transient mechanical deformation of the antenna within a stressed membrane environment. However, these perturbations relax rapidly. qH, by contrast, persists for hours or even days, suggesting a different stabilizing mechanism—one that must outlast the primary trigger. LCNP, a highly hydrophobic 8-stranded β-barrel lipid-binding lipocalin, is structurally adapted to associate with hydrophobic ligands, such as oxidized fatty acids, truncated acyl chains, and stress-induced apolar metabolites (Charron et al. 2005; Malnoë et al. 2018; Dong et al. 2024). Lipocalins often associate with membrane interfaces, modulating packing density, curvature, and lateral pressure. Under chronic high light, thylakoid membranes accumulate de-epoxidized xanthophylls (Demmig-Adams and Adams 1992) and oxidized galactolipids, monocalactosyldiacylglycerol (MGDG) and digalactosyldiacylglycerol (DGDG), that alter bilayer viscosity and thickness, properties that determine the strength of hydrophobic mismatch.

I propose that LCNP binds lipids surrounding LHCII and stabilizes the mismatch-enforcing environment. In this model, LHCII enters a mismatch-induced quenched state during NPQ; with persistent stress, LCNP accumulates and “locks in” the membrane deformation, converting short-lived mismatch into sustained qH. The protein ROQH1 acting as an accelerator of qH relaxation (Amstutz et al. 2020) could simply restore the mismatch caused by LCNP by interacting with the latter and releasing the lipids. This mechanism explains: (i) ΔpH independence of qH; (ii) slow induction/relaxation kinetics; and (iii) antagonism by SOQ1. It also suggests a strategy for experimental testing: isolation of LCNP-containing complexes from thylakoid membrane in light harvesting and dissipative states and analysis of lipid composition (as in Wilson et al. 2024); stabilization of low-fluorescence states upon reconstitution of LHCII with LCNP in artificial membranes; and loss of qH in LCNP pocket mutants. By integrating LCNP biology with the hydrophobic-mismatch framework, this model proposes that transient and sustained NPQ arise from the same underlying physical principle: membrane–protein mechanical coupling acting over different timescales. In this view, qH represents a mechanically stabilized version of quenching in LHCII, placing both processes along a continuum of membrane–protein interactions.
